# Gouty Toes and Rosacea Nose: Does Enlightenment-Era Art Suggest a Correlation?

**DOI:** 10.7759/cureus.51438

**Published:** 2024-01-01

**Authors:** Mohammed Abrahim

**Affiliations:** 1 Division of Emergency Medicine, Department of Family Medicine, McMaster University, Hamilton, CAN

**Keywords:** medical art, uric acid, rhinophyma, podagra, rheumatic diseases, history of medicine, rosacea, gout

## Abstract

Gout, one of the most ancient documented diseases in history, has long captivated artists, yielding a rich collection of artworks. This interest peaked during the Enlightenment era in Europe, a time marked by a surge in gout cases alongside rising wealth, consumerism, and subsequent increased public access to artists. This editorial aims to highlight an intriguing observation of a novel association within several Enlightenment-era paintings depicting individuals suffering from gout and often also portraying the distinctive red noses and cheeks seen in patients with rosacea. Traditionally, both rosacea and gout have been classified as localized inflammatory diseases. However, recent studies challenge this conventional categorization, suggesting that these conditions might be components of systemic inflammatory syndromes. Despite the widespread prevalence of these conditions, their potential interconnectedness and shared pathophysiological pathways remain unexplored. Therefore, the representation of gout and rosacea in historical art could extend beyond mere artistic interest, offering a unique and critical perspective for contemporary medical research.

## Editorial

Gout occupies a unique place in the history of medical art, transcending its identity from a mere painful crystal-induced arthropathy to embody a symbol of success, intelligence, and luxury. This transformation can be attributed to its deep-seated association with the unrestrained indulgences of the affluent, encompassing the consumption of rich foods, alcohol, and even engagement in sexual intercourse. The connection between this ailment and the rich and famous has intensified curiosity among both the general population and artists [1].

The Enlightenment era in Europe, characterized by an upsurge in consumerism and the rise of the middle class aspiring to emulate the higher classes, witnessed a profound transformation. While historians may debate the precise delineation of this era, it primarily spanned the seventeenth and eighteenth centuries [1]. Gout, being a symbol of affluence, prompted a proliferation of artistic depictions featuring individuals afflicted by this condition. This period also witnessed increased accessibility to artists and the growing ability of the middle class to commission artworks, including satirical paintings, inadvertently contributing to the glorification of gout. Paradoxically, this excruciating ailment became an unusual badge of honor among the elite [1]. 

**Figure 1:**
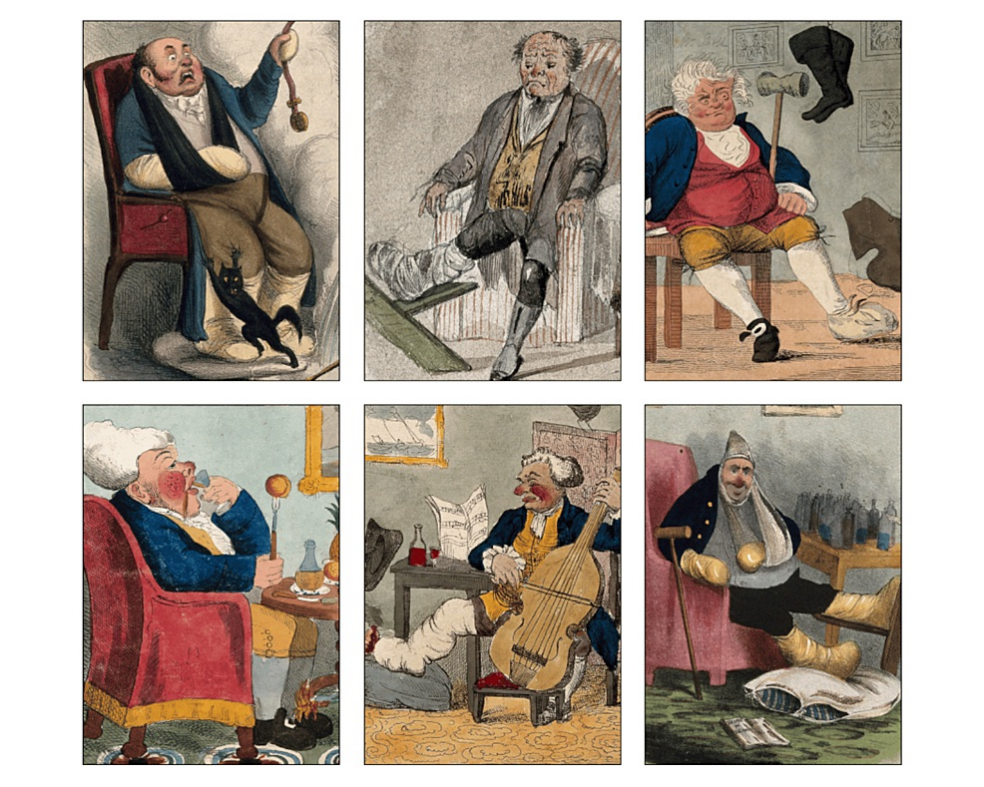
A meticulously composed collage featuring selectively cropped portraits from Enlightenment-era paintings. This collection distinctly showcases individuals diagnosed with gout with prominent red noses and cheeks closely resembling the facial manifestations of rosacea. Public Domain Mark. Source: Wellcome Collection. https://wellcomecollection.org/ [2]. License: https://creativecommons.org/public-domain/pdm/.

It is noteworthy that within this artistic corpus, some depictions of individuals afflicted by gout exhibited facial redness resembling that observed in patients with rosacea, as illustrated in the accompanying collage (Figure 1) featuring six gout patients with rosacea for visual reference [2].

Historically, gout is recognized as the oldest and most prevalent joint disorder affecting humans. Gout is the clinical manifestation of the deposition of monosodium urate crystals in the joints, periarticular soft tissues, and, occasionally, other organs. Gout exhibits a specific affinity for the foot, particularly targeting the first metatarsophalangeal joint. This condition is characterized by intensely painful swelling, manifesting as attacks often triggered by various factors [3].

Rosacea is a poorly understood chronic inflammatory disease of the skin and superficial veins involving primarily the nose and the cheeks and occasionally the eyelids characterized by central facial erythema. Rosacea occurs in phases beginning with dermal flushing, persistent erythema, inflammatory papules/pustules, and telangiectasia (spider veins). While both genders are impacted equally by rosacea, severe forms appear to occur more often in men and involve phymatous changes (rhinophyma) [4]. Emerging evidence suggests that rosacea may transcend its classification as a localized facial skin disorder and could potentially be part of a broader systemic inflammatory syndrome, possibly linked to conditions like metabolic syndrome [4].

In the current state of research, there is a paucity of direct investigations examining the potential link between gout and rosacea. Existing studies have primarily focused on evaluating serum uric acid levels in individuals with rosacea. A notable contribution in this area is the work of Karaosmanoglu et al., who found a statistically significant association between rosacea and hyperuricemia, excluding those with a pre-existing diagnosis of gout. This finding is particularly significant, although it should be noted that the levels of serum urate did not demonstrate a correlation with the severity of rosacea symptoms [5]. In contrast, research conducted by Turkmen indicated that patients with rosacea had significantly lower serum uric acid levels compared to controls [6]. These contrasting findings highlight the complex relationship between gout and serum uric acid.

While hyperuricemia is recognized as the primary risk factor for the development of gout, it is noteworthy that not all individuals with hyperuricemia progress to manifest clinical symptoms of gout. The majority of those with hyperuricemia never experience gout symptoms [7]. Additionally, the initial onset of gout may occur even when serum uric acid levels are within normal ranges [7]. There is also considerable variability in the duration between the onset of asymptomatic hyperuricemia and the clinical manifestation of gout, with this transition period varying widely among individuals, sometimes extending over several years [7]. This complexity in the diagnosis and progression of gout may contribute to the observed discrepancies in the literature regarding serum uric acid levels in patients with Rosacea.

Parallels between rosacea and gout

​​The term gout finds its origins in the French language, as initially introduced in the thirteenth century [3]. Similarly, in French, rosacea is referred to as "gotterose." [4]. Intriguingly, both gout and "gotterose" share a common etymological root in the Latin word "gutta," signifying "drop."

It is noted that both conditions may be exacerbated by the consumption of specific foods and beverages, including alcohol, as well as by environmental and lifestyle factors such as temperature fluctuations and physical exercise [3,4]. Moreover, both gout and rosacea are characterized by recurrent flare-ups. In a recent comprehensive systematic review, rosacea was observed to impact both genders with comparable frequency. However, it is noteworthy that the severe form of rosacea, particularly characterized by phymatous changes in the nasal region (rhinophyma), occurs more frequently among adult men [4]. Similarly, gout, particularly its severe form, is more common in adult men [3]. Additionally, both conditions are notably rare in young individuals and typically manifest between the ages of 30 and 50, with a higher incidence among adults. Furthermore, gout and rosacea primarily affect peripheral or acral regions of the body, although gout can also affect the nose and face. Similarly, gout was originally considered a localized disease, specifically affecting the foot (referred to as podagra). However, it is now firmly established as a systemic condition that can potentially involve a wide range of joints and organs [3,4]. It's noteworthy that obesity and metabolic syndrome are commonly associated conditions in both gout and rosacea [8]. Interestingly, the six individuals depicted in Figure 1 also exhibit signs of obesity, further highlighting the potential links between these conditions.

To conclude, the relationship between gout and rosacea, as portrayed in some Enlightenment-era artworks represents an underexplored domain within academic research. This thematic intersection warrants rigorous scientific examination to either confirm or disprove the potential association between these two conditions. Establishing such a connection could be instrumental in uncovering the etiological underpinnings of both disorders. It is imperative to exercise caution when interpreting historical artworks through a modern medical perspective, due to inherent limitations. These artistic depictions may not consistently align with medical accuracy, as they could be shaped by the artist's perceptions, stylistic choices, or the prevailing cultural milieu of their era. Furthermore, it is essential to avoid the retroactive application of contemporary medical knowledge to past artworks without robust supporting evidence, as this could lead to misinterpretations.
